# Niche Filtering of Bacteria in Soil and Rock Habitats of the Colorado Plateau Desert, Utah, USA

**DOI:** 10.3389/fmicb.2016.01489

**Published:** 2016-09-26

**Authors:** Kevin C. Lee, Stephen D. J. Archer, Rachel H. Boyle, Donnabella C. Lacap-Bugler, Jayne Belnap, Stephen B. Pointing

**Affiliations:** ^1^Institute for Applied Ecology New Zealand, School of Science, Auckland University of Technology, AucklandNew Zealand; ^2^U.S. Geological Survey, Southwest Biological Science Center, Moab, UTUSA; ^3^Institute of Nature and Environmental Technology, Kanazawa University, KanazawaJapan

**Keywords:** biological soil crust, cryptoendolith, Cyanobacteria, desert, Utah

## Abstract

A common feature of microbial colonization in deserts is biological soil crusts (BSCs), and these comprise a complex community dominated by Cyanobacteria. Rock substrates, particularly sandstone, are also colonized by microbial communities. These are separated by bare sandy soil that also supports microbial colonization. Here we report a high-throughput sequencing study of BSC and cryptoendolith plus adjacent bare soil communities in the Colorado Plateau Desert, Utah, USA. Bare soils supported a community with low levels of recoverable DNA and high evenness, whilst BSC yielded relatively high recoverable DNA, and reduced evenness compared to bare soil due to specialized crust taxa. The cryptoendolithic community displayed the greatest evenness but the lowest diversity, reflecting the highly specialized nature of these communities. A strong substrate-dependent pattern of community assembly was observed, and in particular cyanobacterial taxa were distinct. Soils were virtually devoid of photoautotrophic signatures, BSC was dominated by a closely related group of *Microcoleus/Phormidium* taxa, whilst cryptoendolithic colonization in sandstone supported almost exclusively a single genus, *Chroococcidiopsis*. We interpret this as strong evidence for niche filtering of taxa in communities. Local inter-niche recruitment of photoautotrophs may therefore be limited and so communities likely depend significantly on cyanobacterial recruitment from distant sources of similar substrate. We discuss the implication of this finding in terms of conservation and management of desert microbiota.

## Introduction

Terrestrial ecosystems exposed to prolonged moisture deficit are known as deserts or drylands, and they comprise the largest terrestrial biome (Laity, 2008). They are categorized in terms of temperature and aridity, such that hot, cool, and polar deserts are differentiated (Peel and Finlayson, 2007). Deserts are defined as tropical sub-humid, semi-arid, arid, or hyper-arid depending on the level of moisture deficit in the system (UNEP, 1992). A major feature of all deserts is the microbial communities that develop in soil as biological soil crusts (BSC) or as cryptoendoliths within porous rocks such as sandstone (Pointing and Belnap, 2012; Makhalanyane et al., 2015).

Several studies have revealed the diversity and functionality of BSC in deserts (Belnap et al., 2003; Nagy et al., 2005; Belnap, 2006; Gundlapally and Garcia-Pichel, 2006; Bowker, 2007; Bowling et al., 2011; Castillo-Monroy et al., 2011; Maestre et al., 2011; Rajeev et al., 2013), as well as their critical importance to global carbon and nitrogen budgets (Elbert et al., 2012). While moss and lichen are important components of BSC, Cyanobacteria underpin productivity in this system and keystone taxa are largely from the genus *Microcoleus* (Belnap et al., 2003).

Bulk subsurface soils and cryptoendoliths have received comparatively less attention. A limited number of studies has revealed desert soils are dominated by Actinobacteria and Proteobacteria (Navarro-Gonzalez et al., 2003; Chanal et al., 2006; Drees et al., 2006; Lester et al., 2007; Stomeo et al., 2013), and a similar pattern occurs in polar deserts (Pointing et al., 2009; Lee et al., 2012). The diversity of bacteria in cryptoendolithics is poorly understood for all deserts. Microscopy has led to the hypothesis that cryptoendoliths in non-polar deserts are dominated by the cyanobacterial genus *Chroococcidiopsis* (Friedmann, 1980; Büdel and Wessels, 1991; Bell, 1993). However, molecular analysis to further reveal community diversity is lacking for cryptoendoliths in other types of desert.

A surprising finding in polar deserts was that surface soil and rock communities in close proximity shared little similarity and were dominated by different bacterial phyla (Pointing et al., 2009). We therefore set out to test if the same pattern occurs in a cool desert. Here we report a high-throughput sequencing study of adjacent bulk subsurface soil, BSC and cryptoendolith communities in the Colorado Plateau Desert. We reveal evidence for strong niche filtering of photoautotrophic taxa in communities located meters apart, and discuss the implication this has for resilience of cool desert microbial communities.

## Materials and Methods

### Sample Recovery

All samples were recovered during October 2014 from the Colorado Plateau Desert, Utah, USA in the vicinity of Moab, UT, USA (38.538151 N, 109.59623 W). Three locations 100 m apart were selected based on a visual confirmation of co-occurrence for the three substrates. At each location, bulk surface soil (top 50 mm) was recovered directly from a sandy currently dry wash directly into sterile 50 mL centrifuge tubes (*n* = 9, although three samples failed post-sequencing quality control and were excluded from further analysis, thus final *n* = 6). Cryptoendolith was recovered by fracturing sandstone rock with a geological hammer and aseptically collecting visibly colonized fragments in sterile Whirlpak bags (*n* = 9). The BSC samples were excised from locations adjacent to each soil and rock sample aseptically using sterilized scalpel and forceps (*n* = 18) and stored in sterile 50 ml centrifuge tubes. All samples were preserved at -80°C until processed.

### Sequence Acquisition

Total colonized surface (range from 3–5 cm × 3–5 cm) were sampled from each endolithic fraction by physically scratching the friable sandstone surface with sterilized forceps. Each BSC sample was weighed ~1 g. DNA for the cryptoendolithic and soil crust samples was extracted using the PowerMax Soil DNA Isolation kit (Mo Bio Laboratories, Carlsbad, CA, USA) following manufacturer’s protocol. DNA in bare surface soil samples were extracted via the PowerSoil DNA Isolation kit (Mo Bio Laboratories) following manufacturer’s protocol. All extractions were carried out in a laminar flow hood under aspetic conditions. To improve cell lysis efficiency, tubes containing the samples and buffer solution were incubated in a water bath at 65°C for 30 min before the vortex step.

Extracted environmental DNA was resuspended in UltraPure H_2_O and then concentration in each sample adjusted to 5 ng/uL before Illumina MiSeq library preparation as specified by the manufacturer (16S Metagenomic Sequencing Library Preparation Part # 15044223 Rev. B; Illumina, San Diego, CA, USA). Briefly, PCR was conducted with the primer set: PCR1 forward (5′ TCGTCGGCAG CGTCAGATGT GTATAAGAGA CAGCCTACGG GNGGCWGCAG 3′) and PCR1 reverse (5′ GTCTCGTGGG CTCGGAGATG TGTATAAGAG ACAGGACTAC HVGGGTATCT AATCC 3′) with KAPA HiFi Hotstart Readymix (Kapa Biosystems, Wilmington, MA, USA) and the following thermocycling parameters: (1) 95°C for 3 min, (2) 25 cycles of 95°C for 30 s, 55°C for 30 s, 72°C for 30 s, 72°C for 5 min, and (3) holding the samples at 4°C. The amplicons were then indexed using Nextera XT index kit (Illumina) with an additional eight PCR amplification cycles with the same PCR conditions as above. The indexed amplicons were purified and size selected using AMPure XP beads (Beckman-Coulter, Brea, CA, USA) before sequencing on an Illumina Miseq (Illumina) with the 600 cycle V3 chemistry (300 bp paired-end reads). A 5% PhiX spike-in was used, as per manufacturer’s recommendation.

### Bioinformatic Analysis

USEARCH v8.0.1623 (Edgar, 2013) was used to quality control and process the raw sequencing reads, with the following workflow to remove anomalous sequences. First, the forward and reverse paired-end reads were merged, and the merged reads with lengths outside 200–500 bp range or exceeding six homopolymers were removed by Mothur v1.36.1 (Schloss et al., 2009). Next, the sequences were subjected to *Q* score filtering to remove reads with maximum expected error > 1. Singleton reads were then removed.

Representative sequences of the operational taxonomic units (OTUs; Supplementary Table S2) were taxonomically assigned using the RDP classifier (Wang et al., 2007) implemented in QIIME v1.9.1 (Caporaso et al., 2010). Greengenes release 13_8 (McDonald et al., 2012) was used as the reference taxonomic database. The OTU table was rarefied to 16,058 sequences per sample to remove sample heterogeneity; rarefaction analysis indicated that sequencing depth was sufficient to identify the majority of OTUs within each community (Supplementary Figure S1). Rarefaction, alpha and beta diversity analyses were performed in QIIME (Caporaso et al., 2010). Permutational multivariate analysis of variance (PERMANOVA) on the community weighted UNIFRAC distance matrix was performed with the adonis function in the R package “vegan” v2.3.3 (Oksanen et al., 2015); 1000 replicate permutations were made. Differential abundance analysis was performed with DESeq2 (Love et al., 2014) as implemented in the R package “phyloseq” v1.15.2 (McMurdie and Holmes, 2013). All sequence data is available under the study accession number PRJEB15188.

## Results

**FIGURE 1:**
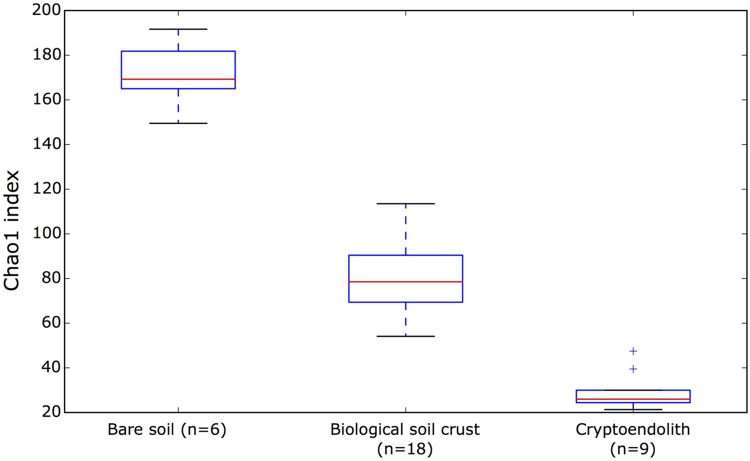
**The estimated alpha diversity of bare surface soil, microbial crust, and cryptoendolith microbial communities with Chao1 index.** For alpha diversity values of individual samples, please refer to Supplementary Table S1.

**FIGURE 2:**
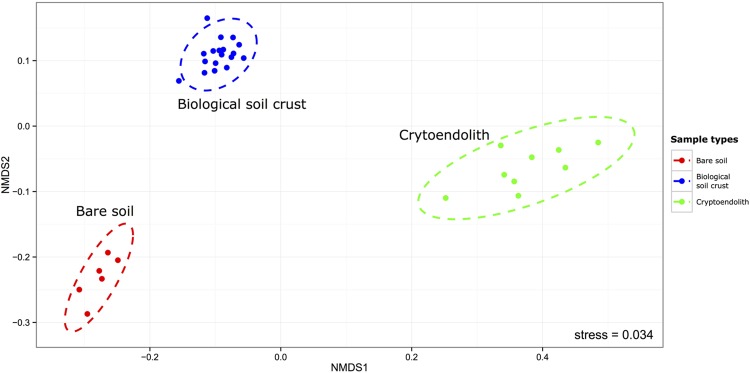
**Non-metric multidimensional scaling plot of Weighted-Unifrac distances between samples.** The ellipses represent 95% confidence from the standard deviations of the points within the sample/microhabitat type (PERMANOVA *R*^2^ = 0.66, *p*-value = 0.001).

This study revealed bacterial diversity in soil and rock substrates of the Colorado Plateau Desert using a high throughput sequenicng approach. Three distinct communities were examined: bare subsurface soil, BSC and a cryptoendolithic community within sandstone. The amount of recoverable DNA was used as a very rough approximation for biomass, and revealed that soil and sandstone supported approximately fourfold less recoverable DNA than BSC. Overall, BSC samples yielded highest DNA concentration with a mean of 34.67 ng/uL. In comparison, endolithic samples yielded 9.66 ng/uL and bare surface soil 8.46 ng/uL. A total of 898,305 reads with 288 million base-pairs (bp) were generated. The median read length was 443 bp. After quality filtering sequences were clustered *de novo* (with a minimum identity of 97%) into 3925 OTUs among the 33 desert samples. The rarefaction analysis of our sequence data indicated sampling was sufficient to be representative of the three communities (Supplementary Figure S1) with an average of 98.9%, and a minimum of 97.96% Good’s estimated coverage (rarefied at 16058 reads) in the sampled communities (Supplementary Table S1). The bare soil supported greatest alpha diversity, followed by BSC and then cryptoendolithic communities (**Figure 1**). The total richness for the study was 3,925 OTUs, revealed from 898,305 quality-filtered reads overall. The greatest number of shared OTUs was between bare soil and BSC, and the least between bare soil and cryptoendolith, whilst the three communities shared 707 OTUs (Supplementary Figure S2). Dissimilarity analysis revealed that all samples from a given substrate clustered closely and formed highly distinct 16S rRNA gene-defined communities (**Figure 2**). This is further supported by PERMANOVA (adonis function in R package “vegan”), which showed strong partitioning of distance matrix variance by substrate types, such that 66% of overall variation was explained by substrate type (*R*^2^ = 0.66, *p*-value = 0.001).

**FIGURE 3:**
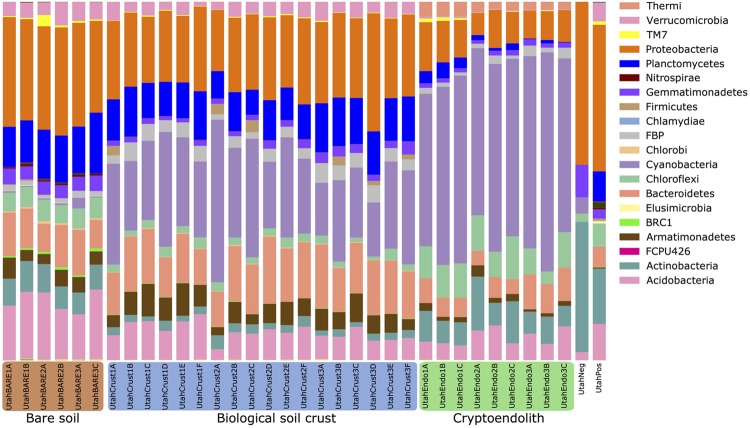
**Relative abundance of prokaryotic phyla/candidate divisions in microbial communities in this study.** Taxa are arranged in the order as they appear on the stacked bar graph. Communities are grouped into type of samples where the communities originated. Low abundance taxa (not visible) include, in Domain *Archaea*: *Crenarchaeota*, *Euryarchaeota*, “Parvarchaeota”; in Domain *Bacteria*: *Fibrobacteres, Tenericutes*, GN02, MVP-21, NKB-19, OD1, OP11, OP3, SR1, TM6, WPS-2, WS2, WS3, ZB3, and BHI80-139. UtahNeg was the negative extraction control with only 44 reads. UtahPos was the positive extraction control from common garden soil.

Overall the study recorded twenty abundant bacterial phyla, plus a rare biosphere of low abundance taxa from 18 additional phyla that were largely candidate divisions (**Figure 3**). Several phyla were ubiquitous across all three communities, notably the Acidobacteria, Bacteroidetes, Cyanobacteria, and Proteobacteria. Bare soil displayed greatest evenness (Supplementary Figure S3) with Proteobacteria being the most abundant phylum, whereas in BSC showed a greatly enhanced abundance in Cyanobacteria was recorded. The cryptoendolithic community was also dominated by Cyanobacteria, although dominant OTUs were from different genera in cryptoendolithic communities (*Chroococcidiopsis*) vs. BSCs (*Phormidium/Microcoleus* group; Supplementary Figure S4). The cryptoendolith also displayed elevated relative abundance of Actinobacteria and Chloroflexi, with relatively lower abundance for all other phyla. Interestingly, despite the overall dominance of Cyanobacteria, this phylum was less diverse (i.e., fewer cyanobacterial OTUs) than those of other phyla (Supplementary Table S3), suggesting relatively low phylogenetic diversity for the Cyanobacteria within the community.

## Discussion

This study illustrates how desert micro-habitats in close proximity can support distinct microbial communities, and mirrors a similar observation for surface soil and rock habitats in a polar desert (Pointing et al., 2009). This trend likely reflects animal and plant ecology in all deserts where niche-filtering by the physical environment is an important driver of communty assembly (Chase and Leibold, 2003; Maestre et al., 2012; Pointing et al., 2015). The most obvious difference occurred between bare soil and the two developed microbial communities in BSC and cryptoendolithic habitats. It is clear that bare soils are dominated by heterotrophic taxa, whereas BSC and lithic substrates are dominated by Cyanobacteria and are therefore most likely driven by photoautotrophy. As such the latter are major sites of productivity in extreme drylands (Pointing and Belnap, 2012; Pointing et al., 2015) and estimates suggest they contribute significantly to global productivity (Elbert et al., 2012).

Bare soils experience greater fluctuation in abiotic variables and are relatively unstable as a substrate (Pointing and Belnap, 2012). Quantitative estimates of biomass have demonstrated that bacterial abundance can be several orders of magnitude lower than in surrounding lithic substrates (Pointing et al., 2009), and this also applies to archaea and eukaryotic microorganisms (Wong et al., 2010). The combination of low abundance and a relatively large ‘rare biosphere’ component in desert soil communities suggests that bare soil may not be a significant source of biogeochemical transformations (Fuhrman, 2009), although the higher diversity does imply a potential for greater multifunctionality in soils (Delgado-Baquerizo et al., 2016). Whilst the soil bacterial diversity estimates likely include a large number of inactive taxa, the large number of shared OTUs we observed indicates they may be an important reservoir for recruitment to BSC and cryptoendolith. Indeed, other studies in hot (Makhalanyane et al., 2012) and polar deserts (Wei et al., in review) also indicate that inter-niche bacterial recruitment may occur in extreme deserts. This may become particularly significant in situations where local recruitment is important after disturbance (Belnap and Eldridge, 2003; Kuske et al., 2012) as well as leading to dispersal over large distances (Pointing and Belnap, 2014).

In contrast to bare soil, the development of BSC results in a highly productive community with a significant role in carbon and nitrogen fixation (Delgado-Baquerizo et al., 2010; Bowker et al., 2011; Elbert et al., 2012) as well as soil stability (Belnap, 2003). This is in part due to the more complex spatial structure in BSC (Belnap et al., 2003), and the associated adaptations such as UV protective compounds (Bowker et al., 2002; Gao and Garcia-Pichel, 2011), hygroscopic extracellular polymeric secretions (Mazor et al., 1996; Billi and Potts, 2002) and diel migrations that leave polysaccharides throughout the soil surface (Garcia-Pichel and Pringault, 2001; Rajeev et al., 2013).

The cryptoendolithic community has only recently emerged as a major source of standing biomass in deserts (Pointing and Belnap, 2012). It is interesting that the cyanobacterial taxa in this community are fundamentally different from those in BSC. The cryptoendolithic specialist *Chroococcidiopsis* has relatively small coccoid cells and a slow growth rate, it is well adapted to penetrating pore spaces in the sandstone matrix with little competition, as well as producing copious extracellular polymeric secretions that are implicated in moisture retention in exposed rocky substrates (Chan et al., 2012; Pointing, 2016). Outside the rock it is seen as a poor competitor against faster growing filamentous taxa that also possess UV-tolerance, faster growth and high motility mechanisms that may also confer a competitive advantage (Gao and Garcia-Pichel, 2011). This clear difference in Cyanobacteria between BSC and cryptoendolith suggests these two communities do not interact and cross-recruitment of photoautotrophs does not occur.

The enhanced abundance of Chloroflexi in cryptoendoliths relative to other substrates may be due to the light regime and the photoheterotrophic nature of this phylum. The Chloroflexi can also form a major part of the phototrophic assemblage in hypolithic (Lacap et al., 2011) and geothermal (Lau et al., 2006) settings at the expense of cyanobacterial abundance. Because few physical impediments to cryptoendolithic colonization are envisaged for bacteria, the differences observed in bacteria between BSC and cryptoendolithic communities are likey a reflection of selective forces other than porosity. This is in contrast to eukaryotic taxa that, presumably due to size constraints, display strong porosity-driven selection in cryptoendoliths (De Los Rios et al., 2004, 2007; Wong et al., 2010).

Overall our study has demonstrates that bare soil, BSC and cryptoendolith support distinct bacterial communities in close spatial proximity. The differences in keystone taxa for each reflect the strong niche filtering between substrates. The threatened status of well-developed BSC in drylands due to climate change (Belnap et al., 2008) and disturbance (Belnap and Eldridge, 2003) is a growing concern because of slow recovery rates (Belnap and Eldridge, 2003) and this may impact longterm resilience (Kuske et al., 2012; Steven et al., 2012). Our study indicates that local recruitment from other niches may occur for heterotrophs but that photoautotrophic taxa in BSC and cryptoendoliths likely rely on recruitment from the same substrate either locally or via long- distance recruitment of propagules, thus emphasizing the need to avoid large scale impact to these fragile communities.

## Author Contributions

JB and SP conceived the study. SP and JB conducted field work. KL, SA, and RB conducted experimental work. KL, DL-B, and SP analysed data. JB and SP wrote the manuscript. All authors read and commented on the draft manuscript.

## Conflict of Interest Statement

The authors declare that the research was conducted in the absence of any commercial or financial relationships that could be construed as a potential conflict of interest.
