# Supplementary material for: Niche Filtering of Bacteria in Soil and Rock Habitats of the Colorado Plateau Desert, Utah, USA
Source: Front Microbiol. 2016 Sep 26;7:1489. doi: 10.3389/fmicb.2016.01489 (PMC5035745; doi:10.3389/fmicb.2016.01489)
Supplement: Supplementary file 2 [file Image_1.PDF]

Supplementary Figures

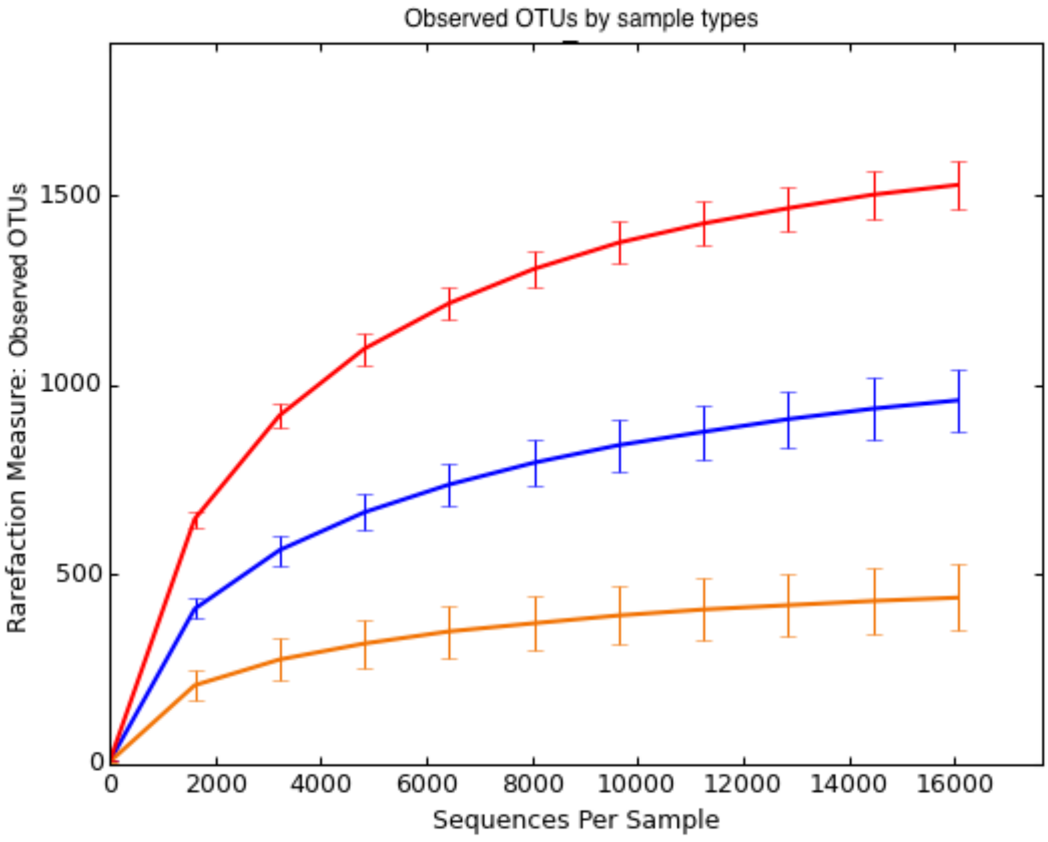

Supplementary Figure 1 – Rarefaction curve showing number of observed OTUs in Utah desert microbial communities, grouped by microhabitat types. The colors indicate: Red - bare soil. Blue – biological soil crust. Orange - cryptoendolith.

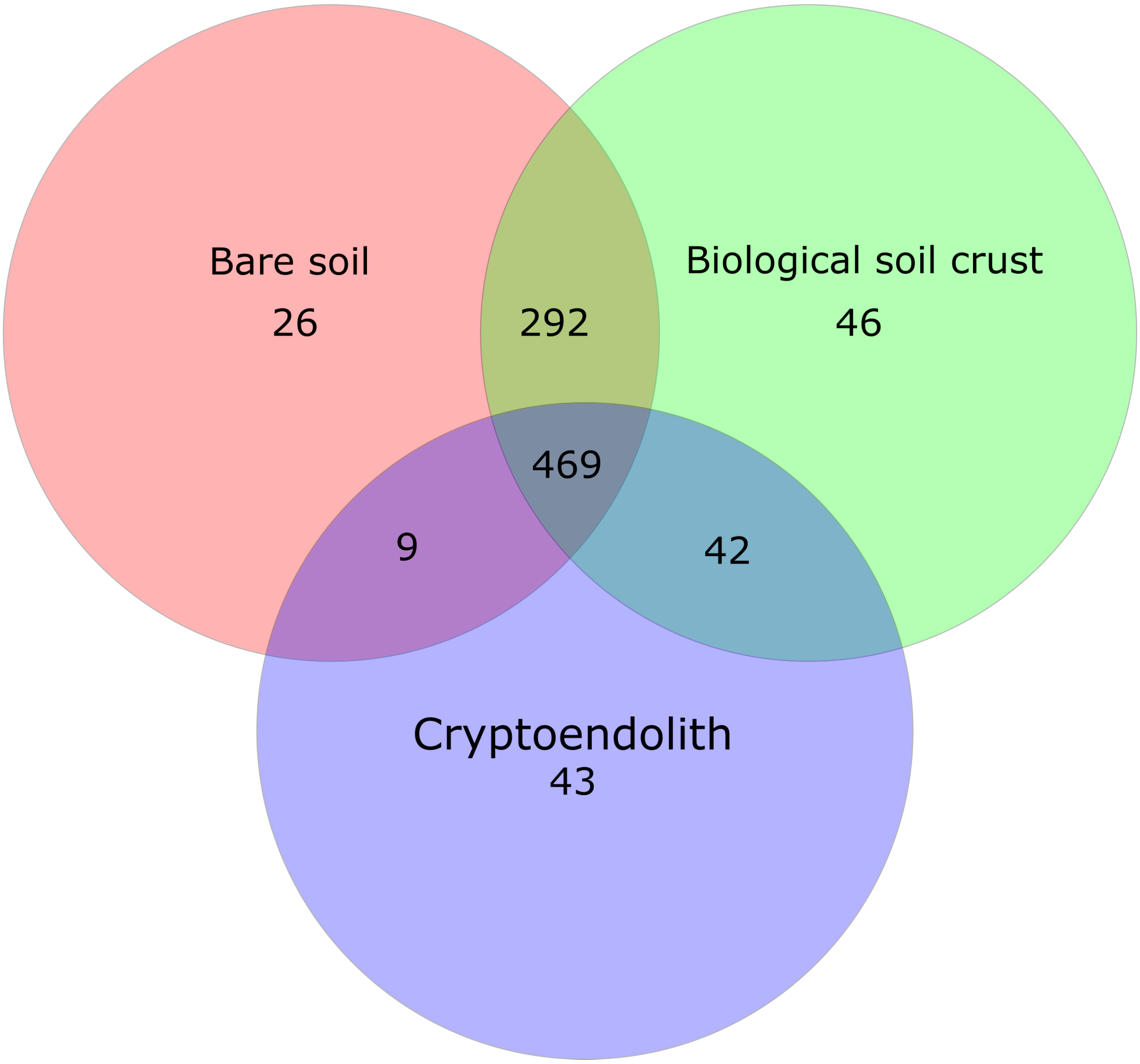

Supplementary Figure 2 – Venn diagram showing shared OTUs between microbial communities in three types of microhabitats. OTUs representing minor taxa with less than 0.01% of total abundance were filtered from this analysis

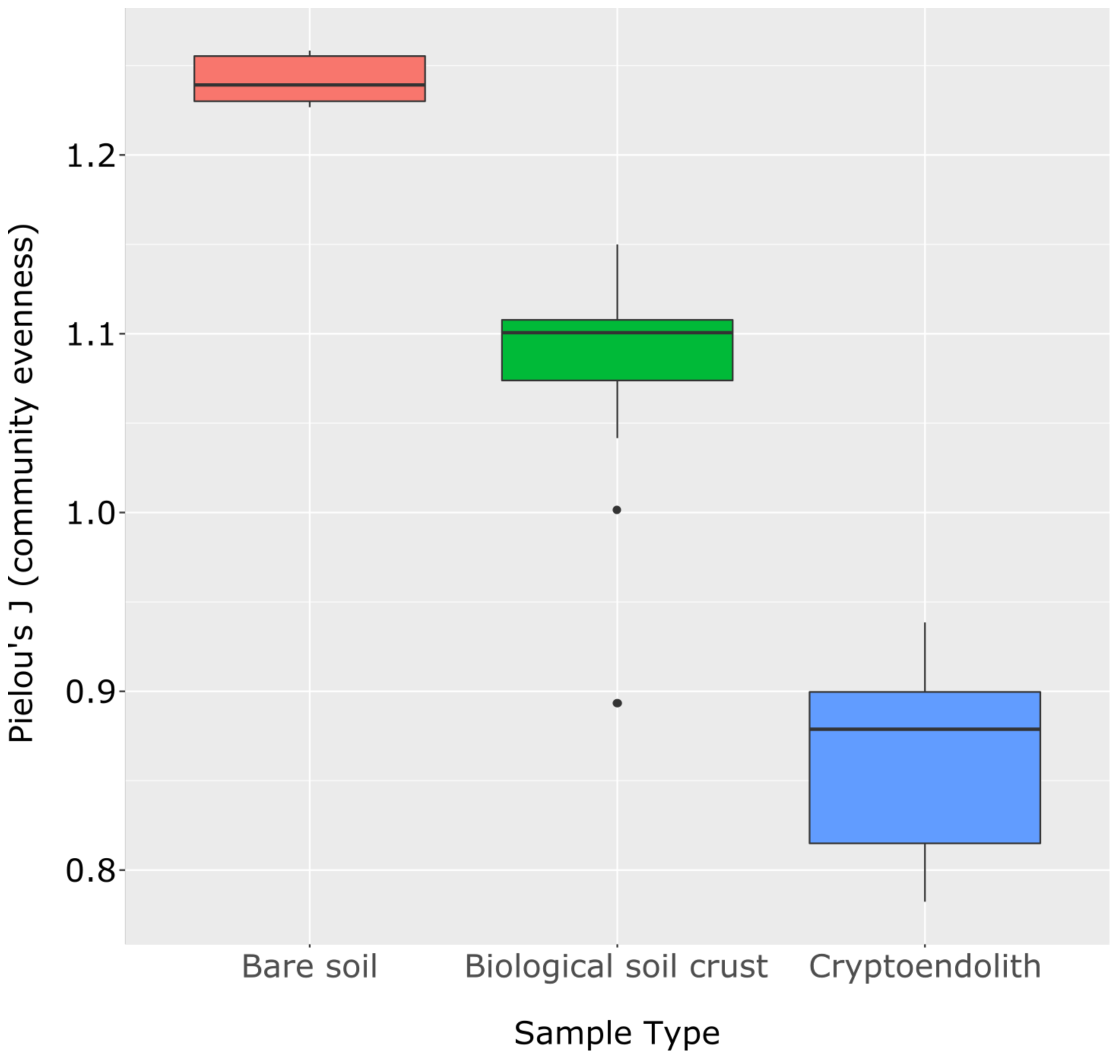

Supplementary Figure 3 – The distribution of community evenness, as measured by Pielou's J, in three microhabitat types.

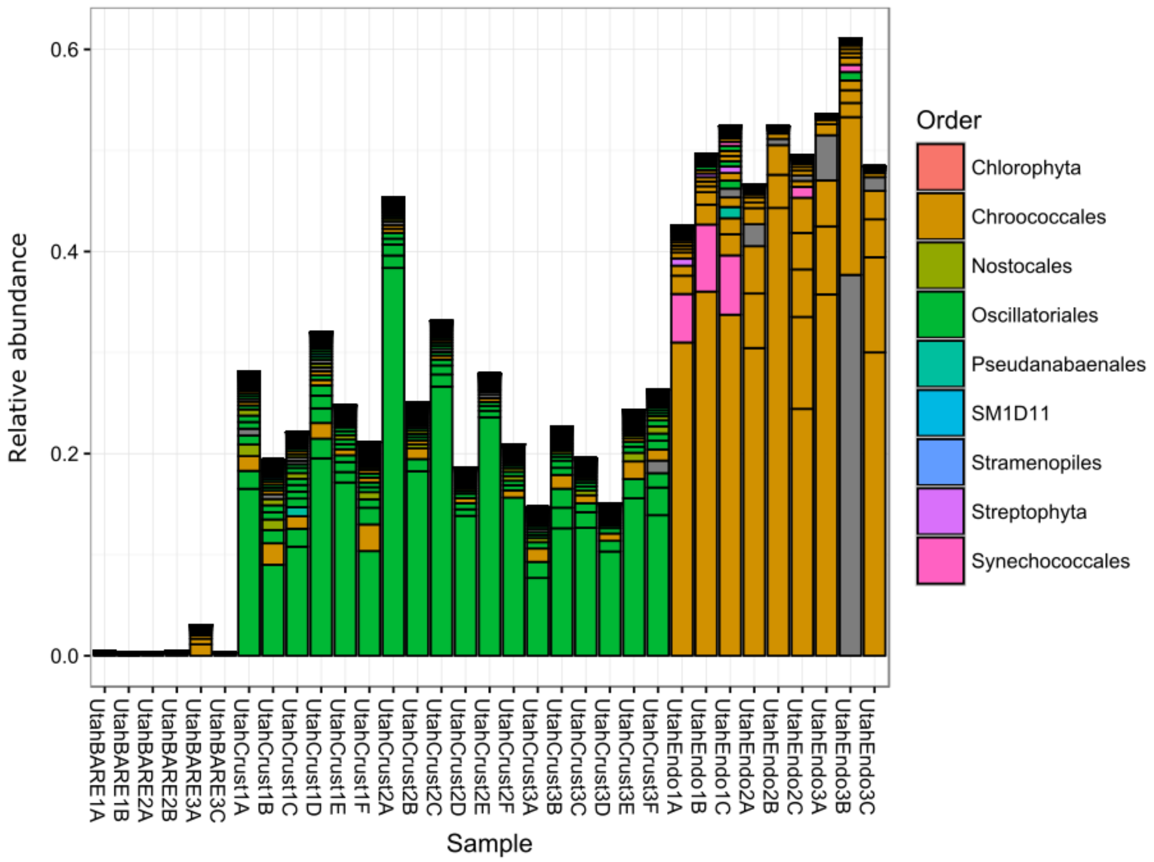

Supplementary Figure 4 – Relative abundance of cyanobacteria and taxa composition. The large grey portion in sample UtahEndo3C represents large number of reads of eukaryotic origins (Chlorophyta).
